# Ocular Trematodiasis in Children, Sri Lanka

**DOI:** 10.3201/eid2904.221517

**Published:** 2023-04

**Authors:** Chandana H. Mallawarachchi, Mangala M. Dissanayake, Sidesh R. Hendavitharana, Saman Senanayake, Nisayuri Gunathilaka, Nilmini T.G.A. Chandrasena, Thishan C. Yahathugoda, Susiji Wickramasinghe, Nilanthi R. de Silva

**Affiliations:** Medical Research Institute, Colombo, Sri Lanka (C.H. Mallawarachchi);; District General Hospital, Trincomalee, Sri Lanka (M.M. Dissanayake, N. Gunathilaka);; Base Hospital, Kantale, Sri Lanka (S.R. Hendavitharana);; National Hospital, Kandy, Sri Lanka (S. Senanayake);; University of Kelaniya, Kelaniya, Sri Lanka (N.T.G.A. Chandrasena, N.R. de Silva);; University of Ruhuna, Galle, Sri Lanka (T.C. Yahathugoda);; University of Peradeniya, Peradeniya, Sri Lanka (S. Wickramasinghe)

**Keywords:** ocular trematodiasis, trematode, parasites, zoonoses, helminths, histopathology, phylogeny, *ITS2* gene, internal transcribed spacer 2, Sri Lanka

## Abstract

Using histopathology and phylogenetic analysis of the internal transcribed spacer 2 gene, we found >2 distinct trematode species that caused ocular trematode infections in children in Sri Lanka. Collaborations between clinicians and parasitologists and community awareness of water-related contamination hazards will promote diagnosis, control, and prevention of ocular trematode infections.

Helminths are major etiologic agents of human blindness in low-income countries (*1*). Adult, juvenile, and larval stages of nematodes, cestodes, and trematodes have been recovered from ocular and periocular tissues. Some helminths are natural parasites of humans, although most are zoonotic (*2*). Most eye helminthiases are accidental, resulting from aberrant migration of immature worms in host tissues. Ocular helminthiases reported in Sri Lanka have been mainly caused by nematode species such as ascarids, filariids, and strongylids (*3*–*7*). Adult avian trematodes (*Philophthalmus* spp.) causing accidental subconjunctival infection in humans have also been reported in Sri Lanka (*5*,*6*). Rare occurrences of trematode-induced conjunctival and anterior chamber granulomas have been reported in South India and Egypt (*8*,*9*). Although adult flukes were not identified in those cases, histologic analysis of excised nodules suggested trematode etiology (*8*,*9*), and molecular methods confirmed a trematode etiology in Egypt (*9*). In the cases from South India, trematode DNA with high sequence similarity to *Procerovum varium* flukes (family Heterophyidae) from fish-eating birds was detected (*10*).

Anterior chamber nodules of the eye of suspected helminth etiology have been noted for almost a decade in the North Central Province of Sri Lanka, and clinical outcomes have varied from complete cures to cataracts and blindness. We report 3 cases of episcleral nodules with confirmed trematode etiology in the Eastern and North Central Provinces of Sri Lanka and describe clinical manifestations, histopathology, and phylogeny of the causative trematode species.

## The Study

We conducted a retrospective study with tissue samples from 3 pediatric patients in Sri Lanka. The samples were referred from the ophthalmology units of the District General Hospital in Trincomalee, National Hospital in Kandy, and Base Hospital in Kantale for molecular analysis at the Department of Parasitology, Faculty of Medicine, University of Peradeniya, Peradeniya, Sri Lanka, during 2020–2021. The Ethics Review Committee of the Medical Research Institute, Colombo, Sri Lanka, approved this study (project no. 26/2020).

**Figure 1:**
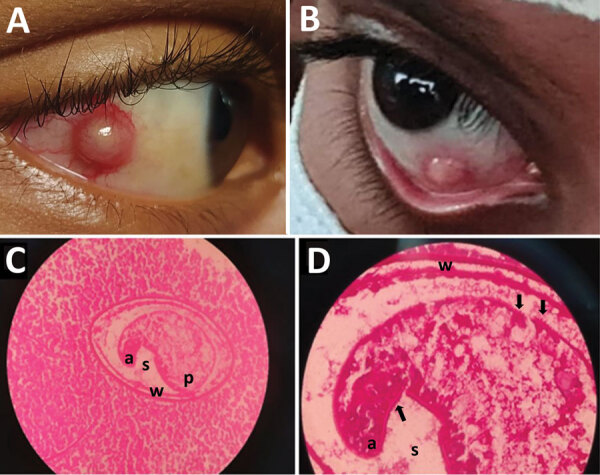
Trematode infection in the eyes of 2 pediatric patients in a study of ocular trematodiasis in children, Sri Lanka. A, B) Episcleral nodules found in eyes of 2 male pediatric patients. C, D) Metacercarial stage of a trematode in hematoxylin/eosin-stained tissue section of an excised episcleral nodule from a 12-year old boy. a, anterior end; p, posterior end; s, space between the larva and cyst wall; w, double layer cyst wall. Arrows in panel D indicate possible spines on surface tegument. Original magnification ×40 for panel C, ×100 for panel D.

In case 1, a 13-year-old male child with a 3-week history of a red right eye in which a nodular lesion developed sought care at the District General Hospital in Trincomalee in September 2020. Examination revealed a dome-shaped, whitish, 3-mm episcleral nodule situated inferonasal ≈3 mm from the limbus (Figure 1). The eye was mildly inflamed, but the patient’s visual acuity was normal. Slit lamp examination did not reveal signs of inflammation in the anterior chamber, and the rest of the affected eye and left eye were normal. The patient was healthy otherwise and had complete blood cell counts and C-reactive protein levels within normal ranges. Several other children living in the neighborhood and 2 siblings of the index case-patient had similar complaints. All affected children had bathed in an irrigation canal connected to the Kantale reservoir.

The nodule was excised under general anesthesia, placed in 10% neutral-buffered formalin, and sent to the pathology laboratory for histopathological assessment, which showed a nodular-shaped granulated tissue fragment with a central cystic area. The cystic area had a micro-abscess with many neutrophils, some eosinophils, and a cross-section of an encysted helminth that was 0.2 mm in diameter (Figure 1). Morphology of the helminth was consistent with the metacercaria stage of a trematode, showing a cyst wall, surface tegument with possible minute surface spines, and a sucker (Figure 1) (*2*). The patient's recovery was uneventful, and no new lesions were observed at a 6-month follow-up examination.

In case 2, a 12-year-old male child from Hingurakgoda in the Polonnaruwa District sought care in April 2021 at the National Hospital in Kandy for a scleral nodule in the right eye that gradually enlarged over a 3-month period (Table). Nodule-associated pain, tenderness, redness, tearing, or impaired vision did not occur. The patient was treated with ocular antimicrobial drugs and steroids but had no improvement. None of his family members had similar complaints. He lived near an irrigation canal connected to the Minneriya reservoir, where he bathed daily. Examination revealed a scleral nodule that was 2 mm in diameter and medial to the limbus of the right eye. The ocular adnexae were normal. A whitish corneal opacity was noted near the limbal border of the nodule (Figure 1).

**Table:** Clinical history of 3 male patients with confirmed ocular trematode infections in study of ocular trematodiasis in children, Sri Lanka*

Case no.	Age, y	Infection site	Clinical manifestations	Trematode identification
1	13	Right eye	A 3-wk history of redness in right eye followed by formation of an episcleral nodule. Examination revealed a dome-shaped, whitish,3-mm episcleral nodule situated inferonasal ≈3 mm from the limbus.	Trematode metacercaria and histologic diagnosis (Figure 1, panels A, B)
2	12	Right eye	A 3-mo history of episcleral nodule in right eye. Examination revealed a scleral nodule, 2 mm in diameter, medial to the limbus.	Unknown trematode isolate 1 and *ITS2* sequencing (Figure 2)
3	11	Right eye	A 3–4-wk history of redness and irritation in right eye and formation of an episcleral nodule. Examination revealed a scleral nodule, 4 mm in diameter, medial to the limbus.	Unknown trematode isolate 2 and *ITS2* sequencing (Figure 2)

*ITS2, internal transcribed spacer 2 gene.

In case 3, an 11-year-old male child from Kantale sought care at the Base Hospital in Kantale for redness and irritation in the right eye in which a nodular lesion developed over a 3–4-week period in December 2020. His visual acuity was normal. A 4-mm episcleral nodule situated medial to the limbus was noted (Table). He also bathed in an irrigation canal connected to the Kantale reservoir.

**Figure 2:**
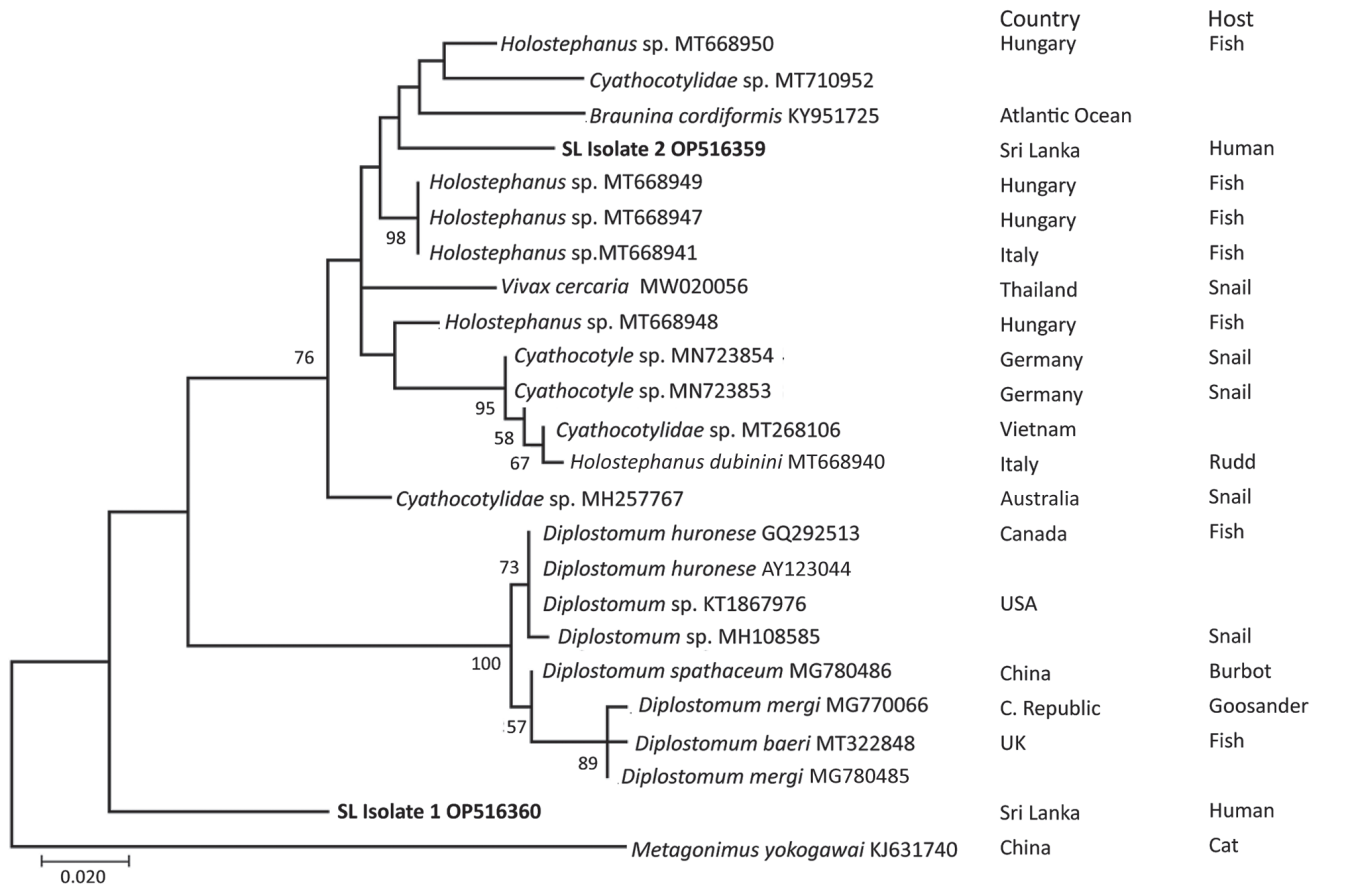
Phylogenetic analysis of isolates from 2 episcleral nodule isolates from the eyes of pediatric patients in study of ocular trematodiasis in children, Sri Lanka. Genomic DNA was isolated from biopsy samples from 2 patients. PCR was used to target the trematode internal transcribed spacer 2 (*ITS2*) gene, which was then sequenced. Maximum-likelihood analysis was used to construct a phylogenetic tree containing partial sequences of isolate 1 and 2 (bold font) from Sri Lanka and 24 taxa from GenBank. Partial *ITS2* gene sequences were aligned by using MEGA version 10.2.4 software (https://www.megasoftware.net). Numbers near nodes indicate the percentages of 1,000 nonparametric bootstrap pseudoreplicates (>50). GenBank accession numbers are provided for *ITS2* gene reference sequences. The sequences for the 2 isolates from this study were deposited in GenBank (accession nos. OP516360 and OP516359). *Metagonimus yokogawai* (KJ631740) isolated from a cat is included as the outgroup. SL, Sri Lanka. Scale bar indicates nucleotide substitutions per site.

We extracted genomic DNA from tissue biopsies (from patients 2 and 3) fixed in 70% ethanol by using the PureLink Genomic DNA Mini Kit (ThermoFisher Scientific, https://www.thermofisher.com). We performed PCR to amplify the internal transcribed spacer 2 (*ITS2*) and 28S rRNA gene regions and mitochondrial *COX1* gene (Appendix). Isolates 1 (patient 2) and 2 (patient 3) were *ITS2*-positive but negative for 28S rRNA and *COX1* by PCR. We performed Sanger sequencing of the *ITS2*-positive samples (Appendix) and constructed a phylogenetic tree by using MEGA-X version 10.2.4 software (https://www.megasoftware.net) and the maximum-likelihood statistical method (Figure 2). The 2 sequences from Sri Lanka that we submitted to GenBank were 487 bp (accession no. OP516359) and 427 bp (accession no. OP516360). Isolate 1 was basal to *Diplostomum* sp., whereas isolate 2 clustered with *Braunina cordiformis* (GenBank accession no. KY951725) (4.4% nt divergence), *Cyathocotylidae* sp. (GenBank accession no. MT710952) (3.8% nt divergence), and *Holostephanaus* sp. (GenBank accession no. MT668950) (1.8% nt divergence) (Figure 2).

## Conclusions

Humans appear to be an accidental intermediate host of the trematodes isolated in Sri Lanka, whereas genomic sequences from trematodes in different countries indicate other hosts, such as snails, fish, birds, and cats. On the basis of our molecular and phylogenetic analyses, we suggest that different trematode species are responsible for ocular trematodiasis in Sri Lanka.

Isolated cases of adult trematode infections of the eye caused by *Philophthalmus* spp., *Fasciola hepatica*, and schistosomes have been reported in other countries (*5*,*6*,*11*–*14*). Trematode cercaria of *Procerovum varium* was proposed as the etiologic agent of ocular inflammatory lesions among children bathing in village ponds in South India (*10*). The route of entry of those cercariae was either oral or by direct penetration of the eye during exposure in snail-infested waters (*10*). *Alaria mesocercaria* trematodes have been reported as a cause of neuroretinitis (*15*). However, infections of human tissues with metacercaria have not been well documented. Our report provides evidence of trematode metacercariae in human ocular tissues. The sequence data indicate that the trematode species found in the isolates from Sri Lanka do not belong to any previously known (or sequenced) species that cause eye infections. The immune-privileged status of the eye might promote cercaria development of the unidentified trematode species in humans.

Excision of the inflamed nodules was curative and enabled extraction of the worm for identification. Establishing the identity of larval helminths in tissue sections is difficult because of rapid tissue degradation caused by substantial inflammation and the requirement for specialist assistance for histopathologic analysis (*7*). Molecular diagnostic methods have overcome these issues, enabling the species-level identification of helminths in tissue sections (*9*,*10*).

In summary, trematode metacercariae might occur in the ocular tissues of children exposed to freshwater reservoirs and their aquatic fauna, posing a water-related public health burden. Ocular trematode infections were caused by >2 distinct trematode species in Sri Lanka. Creating awareness among clinicians and the community and active collaboration with parasitologists will promote diagnosis, control, and prevention of ocular trematode infections.

Appendix Additional information for ocular trematodiasis in children, Sri Lanka.
