# Supplementary material for: Ocular Trematodiasis in Children, Sri Lanka
Source: Emerg Infect Dis. 2023 Apr;29(4):809–13. doi: 10.3201/eid2904.221517 (PMC10045692; doi:10.3201/eid2904.221517)
Supplement: Appendix — Additional information for ocular trematodiasis in children, Sri Lanka. [file 22-1517-Techapp-s1.pdf]

Article DOI: https://doi.org/10.3201/eid2904.221517
EID cannot ensure accessibility for supplementary materials supplied by authors. Readers who have difficulty accessing supplementary content should contact the authors for assistance.

Ocular Trematodiasis in Children, Sri Lanka

Appendix

Additional Methods

Genomic DNA extraction

Genomic DNA was extracted from tissue samples using the PureLink Genomic DNA Mini Kit (ThermoFisher Scientific, https://www.thermofisher.com) according to the manufacturer's protocol. Briefly, samples were cut into small pieces using sterile surgical scissors and pieces were vacuum dried separately for 2–3 min to remove ethanol. Samples were incubated at 55°C for 10 min with proteinase K, RNase A, and genomic lysis/binding buffer, and then 96%–100% ethanol was added to the lysate. The samples were purified with wash buffer using a spin column. Finally, DNA was eluted using genomic elution buffer and stored at −20°C until use.

PCR

We performed PCR to amplify the *ITS2* gene using the universal reverse primer 3S (5′-CGCTGGATCACTCGGCTCGT-3′) and forward primer 28A (5′-CCTGGTTAGTTTCTTTTCCTCCGC-3′). We amplified the 28S rRNA gene by using the forward primer LSU5 (5′-ACCCGCTGAAYTTAAGCA-3′) and reverse primer LSU3 (5′-TCCTGAGGGAAACTTCGG-3′). PCR conditions used to amplify both the *ITS2* and 28S rRNA genes were as follows: initial denaturation at 94°C for 5 min; 30 cycles of denaturation at 94°C for 30 s, annealing at 55°C for 30 s, and extension at 72°C for 1 min; and a final extension at 72°C for 5 min.

The primer pair CF and CR were used to amplify the trematode mitochondrial cytochrome oxidase subunit 1 (*COX1*) gene (*1*). PCR conditions were initial denaturation at 95°C for 4 min followed by 35 cycles of denaturation at 95°C for 15 s, annealing at 54°C for 15 s, and extension at 72°C for 30 s. PCR products were examined by using a 1.5% agarose gel.

References

1. Amin RM, Goweida MB, El Goweini HF, Bedda AM, Lotfy WM, Gaballah AH, et al. Trematodal granulomatous uveitis in paediatric Egyptian patients: a case series. Br J Ophthalmol. 2017;101:999–1002. PubMed https://doi.org/10.1136/bjophthalmol-2017-310259
